# Identification of a Novel *FAM83H* Mutation and Management of Hypocalcified Amelogenesis Imperfecta in Early Childhood

**DOI:** 10.3390/children9030429

**Published:** 2022-03-18

**Authors:** Ji-Soo Song, Yejin Lee, Teo Jeon Shin, Hong-Keun Hyun, Young-Jae Kim, Jung-Wook Kim

**Affiliations:** 1 Department of Pediatric Dentistry, School of Dentistry & DRI, Seoul National University, Seoul 03080, Korea; pedosong@snu.ac.kr (J.-S.S.); lyj72255621@gmail.com (Y.L.); snmc94@snu.ac.kr (T.J.S.); hege1@snu.ac.kr (H.-K.H.); neokarma@snu.ac.kr (Y.-J.K.); 2 Department of Molecular Genetics, School of Dentistry & DRI, Seoul National University, Seoul 03080, Korea

**Keywords:** amelogenesis imperfecta, hypocalcified, general anesthesia, stainless steel crown, zirconia crown, *FAM83H*

## Abstract

Amelogenesis imperfecta (AI) is a heterogeneous group of rare genetic disorders affecting amelogenesis during dental development. Therefore, the molecular genetic etiology of AI can provide information about the nature and progress of the disease. To confirm the genetic etiology of AI in a Korean family with an autosomal dominant inheritance, pedigree and mutational analyses were performed. DNA was isolated from the participating family members and whole-exome sequencing was performed with the DNA sample of the father of the proband. The identified mutation was confirmed by Sanger sequencing. The mutational analysis revealed a novel nonsense mutation in the *FAM83H* gene (NM_198488.5: c.1363C > T, p.(Gln455*)), confirming autosomal dominant hypocalcified AI. Full-mouth restorative treatments of the affected children were performed after the completion of the deciduous dentition. Early diagnosis of AI can be useful for understanding the nature of the disease and for managing the condition and treatment planning.

## 1. Introduction

Amelogenesis imperfecta (AI) is a rare genetic disorder affecting amelogenesis during dental development [1]. The affected enamel can be categorized into three major types: hypoplastic, hypomatured and hypocalcified AI [2]. Hypoplastic enamel is literally thin, in that sometimes it is only a thin covering of unusual mineralization without the normal enamel crystallite structure. The affected enamel is (sometimes extremely) sensitive to cold and hot stimuli, and tooth wear is accelerated after the loss of the thin enamel due to attrition. Wide interdental spacing is a common finding due to the insufficient enamel thickness [3,4]. Hypomatured enamel is less mineralized, due to incomplete removal of the residual enamel matrix proteins or reduced maturation of the enamel crystallite [5]. Dark- to yellow-brown discoloration of the affected enamel is a common finding, and increased attrition and partial fracture of the enamel frequently occur due to hypomineralization [6,7]. Hypocalcified enamel is extremely weak and soft due to improper calcification. The affected enamel is rapidly lost by attrition soon after eruption, leaving a rough and stained enamel [8].

Until now, more than a dozen genes have been identified as causing non-syndromic AI [9,10,11]. Among them, *FAM83H* is the only gene related to the pathogenesis of autosomal dominant hypocalcified AI (ADHCAI) [8,12]. Interestingly, it has been shown that the severe enamel phenotype of ADHCAI, a very weak enamel even weaker than the underlying normal dentin, is not associated with the lack or interruption of the biological function of the wild-type FAM83H in the enamel forming process (amelogenesis) but with the dominant negative effect, even though the exact mechanism of pathogenesis remains to be further elucidated [13,14,15].

Treatment of AI-affected dentition is difficult but important, not only for the oral health, but also for the psychosocial health especially at younger ages [16]. This study aimed to identify a molecular genetic etiology of a family and to report the dental treatments performed on three children in the family.

## 2. Materials and Methods

### 2.1. Family Recruitment

This study was independently reviewed and approved by the institutional review board of the Seoul National University Dental Hospital (SNUDH; IRB No. CRI05003G). Informed consent was obtained from the participating family members for the genetic analysis. Clinical examinations were performed, and blood samples were collected.

### 2.2. DNA Isolation and Whole Exome Sequencing

Genomic DNA was isolated from peripheral blood samples, and the quality and quantity were measured as described before [17]. Whole-exome sequencing was performed with the DNA sample of the father of the proband (III:5) (Theragen Etex, Seoul, Korea). The exome was captured with the Agilent SureSelect Human All Exon Enrichment System, and the 101-bp paired-end sequencing reads were generated using the Illumina HiSeq 2500 (Illumina, Inc., San Diego, CA, USA).

### 2.3. Bioinformatics

The obtained sequencing reads were aligned to the reference human genome assembly (hg38) after trimming for the removal of the adapter sequences. Cutadapt and Burrows–Wheeler aligner were used for the trimming and alignment, respectively [18,19]. A list of sequence variants was obtained after a series of bioinformatics analysis programs, such as Samtools and Genome Analysis Tool Kit [20,21]. Annotation of the variants was performed with the Annovar with dbSNP build 147 [22]. A minor allele frequency of 0.01 was applied as a cutoff value to filter the variants.

### 2.4. Sanger Sequencing

The identified mutation was confirmed by Sanger sequencing with the following primers (713 bp, sense: 5′-ACTTCCTGTCGGCCTTCC-3′; antisense: 5′-GTAGGAGGCCAAACGCC-3′). Sanger sequencing was performed for all three participating family members (Macrogen, Seoul, Korea).

## 3. Results

### 3.1. Mutational Analysis

**Figure 1:**
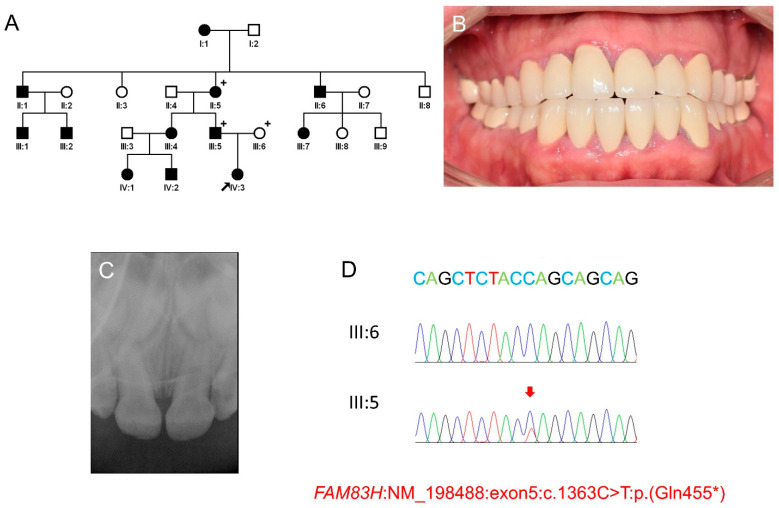
Pedigree, clinical photo of the father, periapical radiograph of the maxillary anterior teeth of the proband, and sequencing chromatograms. (**A**) The pedigree of the family indicates an autosomal dominant inheritance pattern. The plus symbols indicate the participating members for the genetic analysis. A black arrow indicates the proband. (**B**) Clinical photo of the father (III:5) shows a full-mouth restoration. (**C**) A periapical radiograph of the maxillary anterior teeth of the proband at age 2 years 9 months. (**D**) Sequencing chromatograms of the mother (III:6) and the father (III:5). Nucleotide sequence is shown above the chromatogram and the mutated nucleotide is indicated by a red arrow.

Mutational analysis revealed two variations in proven AI-causing genes, *ENAM* and *FAM83H*. The variation in *ENAM* was a missense mutation (NM_031889: c.1348C > T) in the last exon, changing a proline to a serine at codon position 450. In silico predictions gave contradictory results: benign by the SIFT, disease causing by the Mutation taster, and probably damaging by PolyPhen-2 [23,24,25]. Most importantly, the variant was listed in the single nucleotide polymorphism database (dbSNP) with an accession number of rs180899807. The minor allele frequency was as high as 0.027 depending on the study population. The variation in *FAM83H* was a nonsense mutation in exon 5 (NM_198488.5: c.1363C > T) changing a glutamine to an amber stop codon at the 455 position (p.(Gln455*)). This mutation was novel and the nonsense or frameshift mutations in exon 5 in the nearby positions were already proven as ADHCAI-causing mutations [11]. Sanger sequencing confirmed the mutation and segregation within the participating family members (Figure 1). The mutation has been submitted to the ClinVar database (http://www.ncbi.nlm.nih.gov/clinvar, accessed on 18 February 2022) with an accession ID: SCV001976360.

### 3.2. Clinical Phenotype and Treatment of the Proband

At 1 year and 10 months (1Y10M) old, the proband (IV:3) presented to the department of pediatric dentistry, SNUDH, for the management of brown-discolored and hypomineralized dentition. There were no significant problems during pregnancy and delivery, and the family members did not have a remarkable past medical history. The pedigree, clinical photo of the father, periapical radiograph of the proband and sequencing chromatograms are shown in Figure 1. The family history highly suggested autosomal dominant AI and genetic study indeed revealed a novel nonsense *FAM83H* mutation. Therefore, it was anticipated that the extremely soft hypocalcified enamel would be lost and the attrition would be accelerated. Therefore, the treatment for the posterior teeth was planned under general anesthesia (GA). Until the eruption of the deciduous second molars, thorough oral hygiene was instructed, and caution was given about using excessive masticatory force. Additionally, the use of a prevention agent containing casein phosphopeptide-amorphous calcium phosphate (CPP-ACP), GC Tooth Mousse (GC Korea, Seoul, Korea), was recommended. She had a finger sucking habit; therefore, advice for habit control was given.

**Figure 2:**
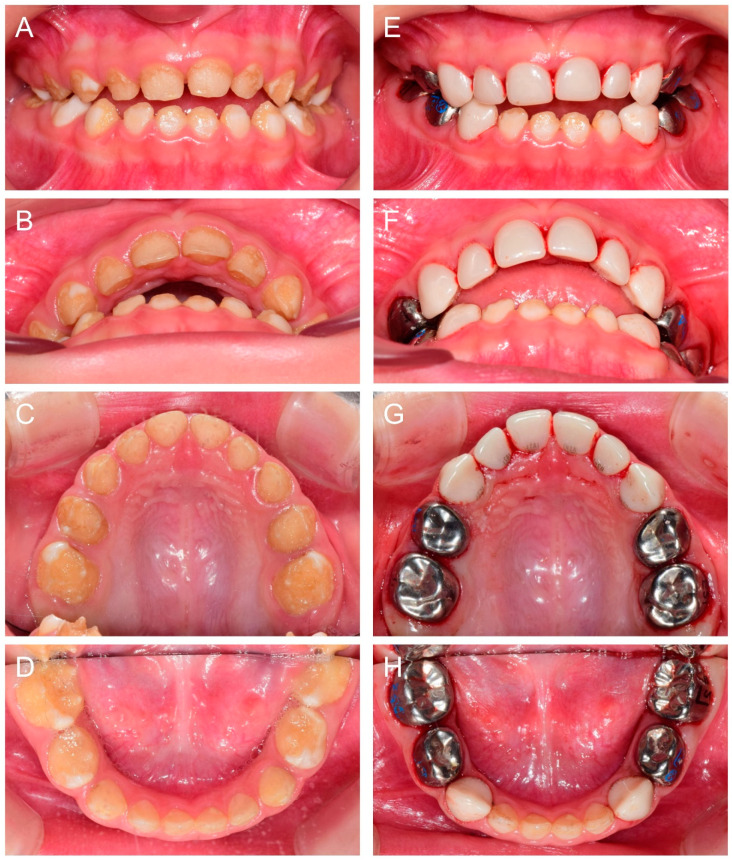
Clinical photos of the proband (IV:3). (**A**–**D**) Clinical photos of the proband before the treatment at age 2 years 9 months. (**E**–**H**) Clinical photos of the proband after the treatment. The deciduous molars were treated with stainless steel crowns, and the deciduous canines and maxillary anterior teeth were treated with zirconia crowns. The deciduous mandibular anterior teeth were left untreated.

**Figure 3:**
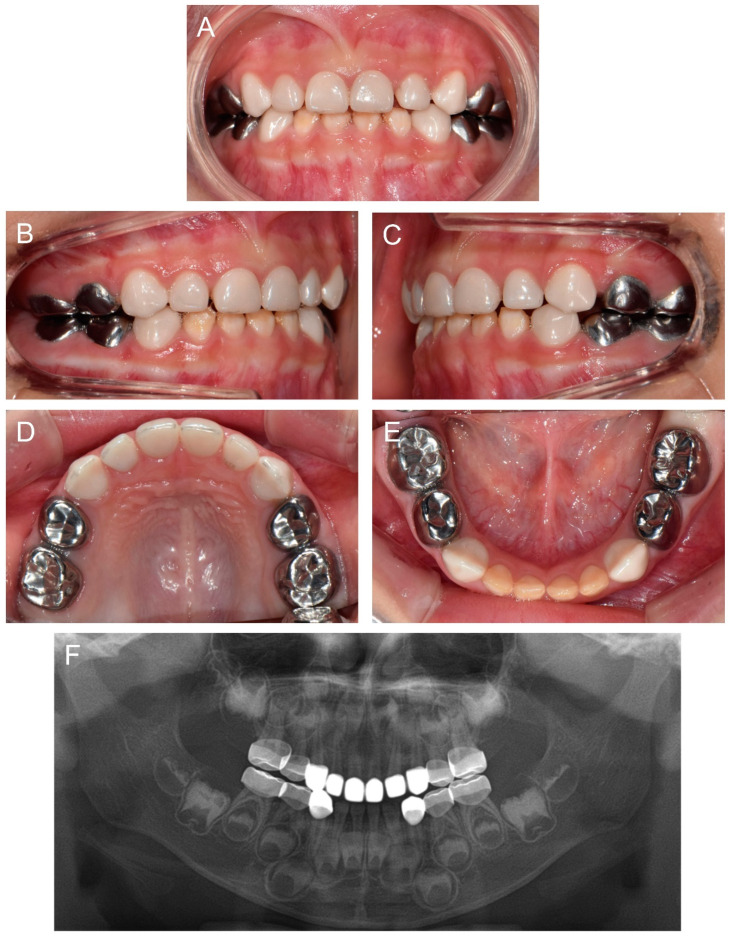
Clinical photos and panoramic radiograph of the proband at age 3 years 11 months. (**A**–**E**) Oral health and restorations were well maintained. Anterior open bite was spontaneously corrected with the discontinuance of the finger sucking habit. (**F**) Panoramic radiograph showed hypocalcified enamel in the developing permanent teeth.

At 2Y9M, full-mouth restoration was performed under outpatient GA (Figure 2). Deciduous molars were treated with stainless steel crowns. Deciduous canines and maxillary anterior teeth were treated with zirconia crowns. Deciduous mandibular anterior teeth were left untreated. At the 3Y11M follow-up (Figure 3), good oral health was maintained, and the anterior open bite was spontaneously corrected with the discontinuance of the finger sucking habit.

### 3.3. Treatment of the Affected Individual (IV:1)

**Figure 4:**
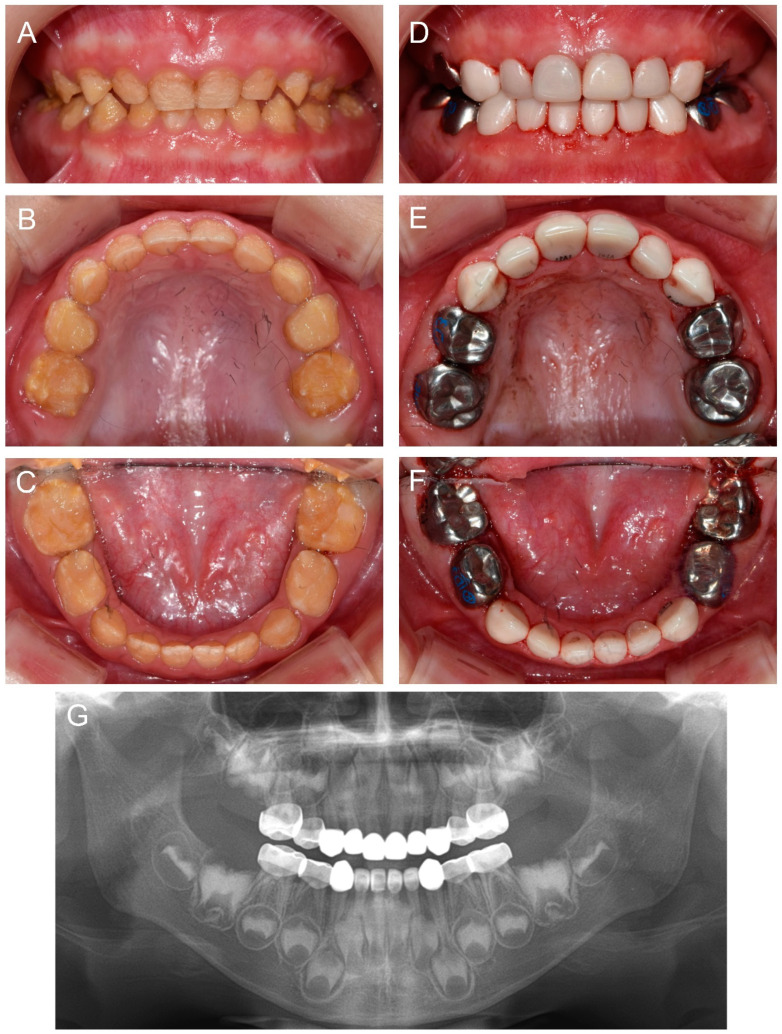
Clinical photos and panoramic radiograph of the affected individual (IV:1). (**A**–**C**) Clinical photos of the proband before the treatment at age 2 years 11 months. (**D**–**F**) Clinical photos of the proband after the treatment. The deciduous molars were treated with stainless steel crowns, and the deciduous canines and maxillary anterior teeth were treated with zirconia crowns. The deciduous mandibular anterior teeth were treated with celluloid resin crowns. (**G**) Panoramic radiograph at age 4 years 9 months.

About 10 months after the first visit of the proband, a cousin of the proband presented for the management of the affected dentition at age 2Y. Oral hygiene instruction was given, and the same preventive measure was recommended. Completion of deciduous dentition was observed at the six-month follow-up, and discomfort in the mandibular posterior area was reported a month later. Full-mouth restoration was performed under outpatient GA at age 2Y11M. The deciduous mandibular anterior teeth were treated with celluloid resin crowns, because there was not enough interdental space for zirconia crowns (Figure 4).

### 3.4. Treatment of the Affected Individual (IV:2)

**Figure 5:**
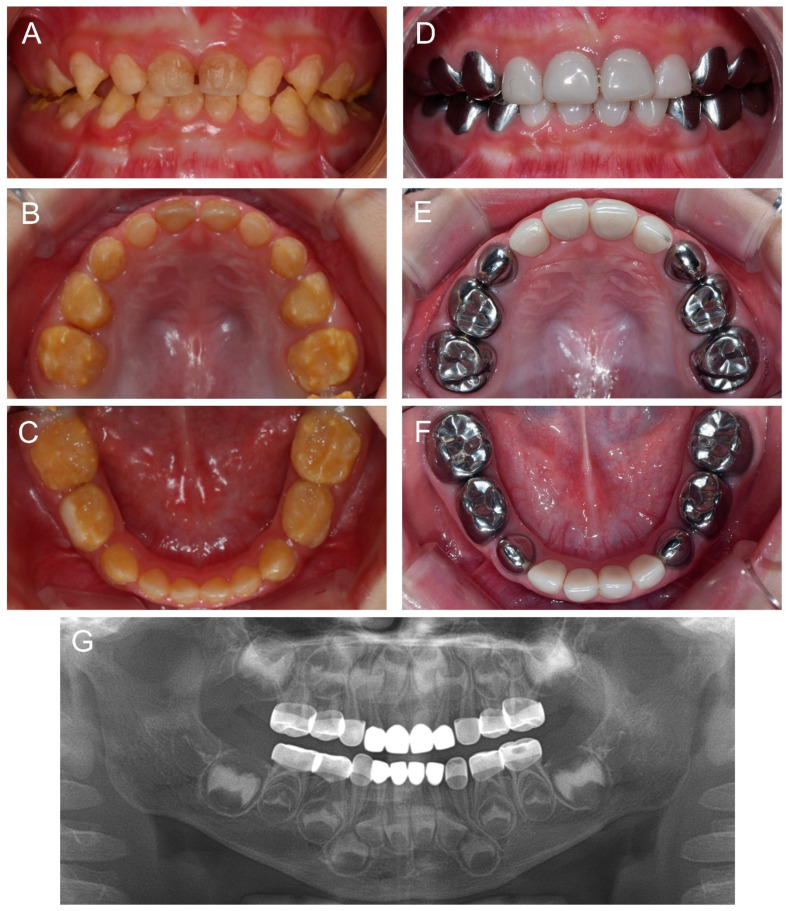
Clinical photos and panoramic radiograph of the affected individual (IV:2). (**A**–**C**) Clinical photos of the proband before the treatment at age 2 years 9 months. (**D**–**F**) Clinical photos of the proband after the treatment at age 3 years 6 months. The deciduous molars and canines were treated with stainless steel crowns and all anterior teeth were treated with zirconia crowns. (**G**) Panoramic radiograph at age 3 years 6 months.

Another cousin (IV:2) of the proband, a younger brother of the affected individual (IV:1), presented for the same reason at age 1Y2M. The same treatment strategy was planned, and full-mouth restoration was performed at age 2Y9M. Deciduous molars and canines were treated with stainless steel crowns and all anterior teeth were treated with zirconia crowns (Figure 5).

## 4. Discussion

Hypocalcified AI is the most frequent form among heterogeneous AI types, and a single gene, *FAM83H*, is responsible for the pathogenesis [1,8]. Therefore, mutations in the *FAM83H* gene could be the most common culprit causing AI. To date, more than 30 mutations have been identified in the *FAM83H* gene [11]. The *FAM83H* gene locates on the long arm of chromosome 8 (8q24.3) and contains five exons. Translation begins from exon 2, and most of the amino acids are encoded by exon 5 (933 out of a total 1179 amino acids). All AI-causing mutations identified to date are nonsense or frameshift mutations occurring in exon 5 (from p.Ser287* to p.Glu694*) [11]. Because of the location in the last exon, the mutation escapes from the surveillance system, nonsense-mediated mRNA decay, and encodes a truncated protein. The C-terminus part seems to be needed for the localization of the wild-type FAM83H in the cytoplasm, even though the functional role is still unclear [15]. Without the C-terminus, the truncated FAM83H moves into the nucleus and is believed to exert a dominant-negative effect disturbing the normal enamel calcification process [14]. It is believed that the novel mutation identified in this study expands the mutational spectrum of the AI-causing *FAM83H* mutation based on the type and location of the mutation (p.(Gln455*)).

It has been shown that AI patients have higher levels of psychosocial problems such as social avoidance and distress in addition to higher levels of dysfunction, discomfort and disability from the affected oral condition compared with individuals without AI [16]. Additionally, they concluded that AI negatively affects the psychosocial health of affected patients to a degree comparable to that of systemic disease, especially at younger ages. Therefore, the treatment for the AI condition is important not only for the prevention of infection or tooth loss but also for improving the psychosocial development or relationship of AI patients by improving their oral health and appearance [26].

The treatment of hypocalcified AI is even more difficult than the other types of AI. Because the enamel calcification itself is not proper, very little help can be obtained from state-of-the-art esthetic resin bonding system. Deproteination from the hypocalcified enamel matrix was suggested to improve the bonding strength; however, long-term stability cannot be guaranteed [27]. If the child is uncooperative or too young to be expected to cooperate during the treatment of sensitive teeth and many teeth need to be treated, treatment under GA can be considered.

In this study, we identified a young patient (proband) with hypomineralized deciduous dentition and found the underlying mutation in the *FAM83H* gene, confirming ADHCAI. It was predicted that the affected enamel would be easily destroyed or lost with attrition or mastication, leaving a rough enamel surface with discoloration and thermal sensitivity [28]. Therefore, full-mouth treatment under GA was planned; however, the primary second molars were not erupted yet. Until the deciduous dentition was complete and ready for treatment under GA, instructions for oral hygiene and precaution to avoid excessive masticatory force were given. Moreover, the CPP-ACP containing agent was recommended to help the mineralization of the hypocalcified enamel or reduce the loss of enamel at least [29]. Even though there is no direct supporting evidence for the hypocalcified enamel, we thought that the calcium and phosphate in the agent would help mineralization if it was used on a daily basis with good oral health [30]. Future studies regarding new or better remineralization agents for hypocalcified enamel are necessary.

Since it has been reported that restoration with crown shows better prognosis than direct composite resin restorations [31], most teeth were treated with zirconia and stainless steel crowns in this study. Long term treatment checkup will be required.

## 5. Conclusions

In this study, a novel *FAM83H* mutation causing ADHCAI was identified and full-mouth treatments of three affected young children were successfully performed under an outpatient GA protocol. If the dentition is not severely affected and the patients are cooperative for the treatment, conventional dental treatment or treatment under sedation could be better treatment options. Dental treatment for the AI condition can prevent infection or tooth loss and improve the psychosocial development in AI patients. In addition to restorative treatment, active preventive measures are also required for AI patients. Further studies would be necessary to find the most effective methods for caries prevention (and/or hypersensitivity reduction) and treatment in AI patients.

## Data Availability

The data presented in this study are openly available in ClinVar (http://www.ncbi.nlm.nih.gov/clinvar, accessed on 18 February 2022), Submission ID: SCV001976360.
